# Use of Ambulance Dispatch Calls for Surveillance of Severe Acute Respiratory Infections

**DOI:** 10.3201/eid2601.181520

**Published:** 2020-01

**Authors:** Susana Monge, Janneke Duijster, Geert Jan Kommer, Jan van de Kassteele, Gé A. Donker, Thomas Krafft, Paul Engelen, Jens P. Valk, Jan de Waard, Jan de Nooij, Wim van der Hoek, Liselotte van Asten

**Affiliations:** European Centre for Disease Prevention and Control, Stockholm, Sweden (S. Monge);; National Institute for Public Health and the Environment, Bilthoven, the Netherlands (S. Monge, J. Duijster, G.J. Kommer, J. van de Kassteele, W. van der Hoek, L. van Asten);; Nivel Primary Care Database, Utrecht, the Netherlands (G.A. Donker);; Maastricht University, Maastricht, the Netherlands (T. Krafft); Meldkamersupport, Hellevoetsluis, the Netherlands (P. Engelen);; Dispatch Center Regional Ambulance Services Noord Nederland, Groningen, the Netherlands (J.P. Valk);; University Medical Center, Groningen (J.P. Valk);; Regional Ambulance Service Hollands Midden, Leiden, the Netherlands (J. de Waard, J. de Nooij)

**Keywords:** influenza, respiratory infections, respiratory syndrome, public health surveillance, emergency medical services, ambulance, the Netherlands, viruses

## Abstract

Ambulance dispatches for respiratory syndromes reflect incidence of influenza-like illness in primary care. Associations are highest in children (15%–34% of respiratory calls attributable to influenza), out-of-office hours (9%), and highest urgency-level calls (9%–11%). Ambulance dispatches might be an additional source of data for severe influenza surveillance.

Influenza virus infection is associated with severe illness and death and causes a high burden of disease (*1*). The World Health Organization recommends that countries improve surveillance of severe influenza infections (*2*). However, surveillance based on hospital and intensive care unit admissions for severe acute respiratory infections or laboratory-confirmed influenza is still limited in most countries, raising the question whether there are other routinely collected data that could be used to monitor severe influenza infections (*2*,*3*).

Ambulance dispatch centers manage patient telephone calls by using a clinical triage system to collect and record information in real time and determine the need for an ambulance and the urgency of the need. This data source is attractive for surveillance because it is recorded continuously, is quickly available, has high coverage within a region, has low running costs, and is highly standardized (*4*–*6*). We aimed to assess whether telephone calls to ambulance dispatch centers were a possible source for surveillance of severe influenza, by showing the association with influenza-like illness (ILI) incidence, the most critical influenza indicator in primary care.

We retrospectively reviewed 289,307 ambulance calls with high urgency (A1 and A2: ambulance required to arrive within 15 or 30 minutes) received in 4 call centers in the Netherlands, covering 4.2 million persons (25% of the population), from January 1, 2014, through December 31, 2016. Therefore, our data covered 2 full respiratory seasons (2014–15 and 2015–16) and 2 half seasons: 2013–14 (weeks 1–26) and 2016–17 (weeks 27–52). Triage diagnostic codes from the Advanced Medical Priority Dispatch System associated with possible respiratory infection were combined as respiratory syndrome calls (RSCs) and aggregated weekly; 6.7% of calls were RSCs (weekly average 123, range 69–165; i.e., 2.9/100,000 inhabitants/wk). RSCs showed a periodic pattern, peaking in winter, with lower interseasonal peaks.

We estimated how many RSCs were attributable to influenza circulation in the community, using weekly ILI incidence from sentinel general-practitioner practices (*7*). We used a binomial generalized linear model with an identity link function to model weekly number of RSCs (numerator) relative to total number of calls (denominator). Model coefficients estimate risk differences; that is, percentage increase in RSCs per each additional case of ILI per 10,000 inhabitants. We evaluated the presence of a linear time trend and periodic patterns and conceptualized it as the baseline (RSCs not attributed to ILI). We then evaluated the association of ILI incidence either in the current week or lagged up to 4 weeks forward in time (+ lags) or 4 weeks backward in time (– lags). Finally, we evaluated whether this association differed between epidemiologic years (week 27 of one year to week 26 of the following year). We repeated the analysis for different age groups, office hours (8:00 AM–16:59 PM Monday–Friday), out-of-office hours (17:00 PM–7:59 AM Monday–Friday, plus the weekend), and calls with the highest urgency level (A1: immediately life-threatening, ambulance required to arrive within 15 minutes).

All models showed a significant positive association (p<0.05) between ILI and RSCs. Increases in RSCs occurred generally 1–3 weeks later than increases in ILI. For patients <15 years of age, RSC increases occurred 1 week earlier than ILI, possibly reflecting respiratory syncytial virus activity rather than influenza activity. Among highest-urgency calls and children, the association varied by epidemiologic year and was highest for 2013–14 and 2016–17.

Overall, an increase in ILI weekly incidence of 1 case/10,000 inhabitants was associated with an increase of 0.114% in weekly RSCs. In our study population, this finding translated into an average of 110 RSCs/year, or 2.6 cases/100,000 inhabitants/year.

When applying these effects to observed weekly ILI incidence (4.98 cases/10,000 inhabitants), we attributed 12.9 RSCs/100,000 inhabitants/year to ILI. The highest proportion of ILI-attributed RSCs was in patients <15 years of age (15%–34%, depending on the season), during out-of-office hours (9%), and highest-urgency calls (9%–11%). In all models, the ILI-attributed percentage was higher during the winter peaks, but non-ILI baseline still explained most RSCs during these winter periods (Figure), showing the expected high background incidence in dispatch data of other respiratory conditions, even during the peak of the influenza season, as also reported by others (*4*,*5*,*8*).

**Figure:**
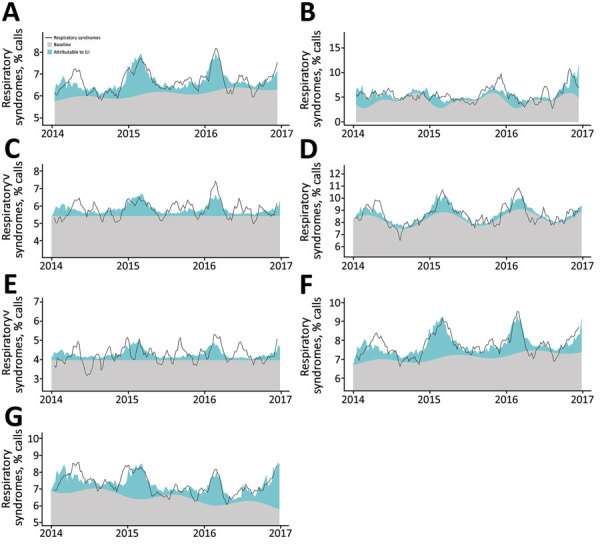
Observed and predicted weekly proportion of ambulance dispatch calls with respiratory syndromes from the multivariate models. The gray area represents the proportion that the model identifies as the baseline (i.e., attributable to unidentified factors); the colored area is the proportion of ambulance dispatch calls with respiratory syndromes attributed to influenza-like illness. The black line is the 5-week moving average of the observed proportion of respiratory syndromes. A) Overall; B) patients <15 years of age; C) patients 15–64 years of age; D) patients >65 years of age; E) calls during office hours; F) calls during out of office hours; G) calls of urgency level A1.

In conclusion, ambulance calls for respiratory syndrome show variability attributable to ILI and probably reflect the severe end of the ILI spectrum, especially when restricted to calls for the young, during out-of-office hours, or with the highest urgency level. Ambulance dispatch center data could be used to monitor the severity of the influenza epidemic, but its true usefulness and added value should be studied prospectively. Such surveillance could potentially inform planning of healthcare services and public health actions such as vaccination, especially following the emergence of new influenza strains. Our results also estimate the impact of influenza on ambulance services and can add to the estimations of burden of disease for influenza.
